# Socioeconomic inequalities in activities of daily living limitations and in the provision of informal and formal care for noninstitutionalized older Brazilians: National Health Survey, 2013

**DOI:** 10.1186/s12939-016-0429-2

**Published:** 2016-11-17

**Authors:** Ma.Fernanda Lima-Costa, Juliana V. M. Mambrini, Sérgio V. Peixoto, Deborah C. Malta, James Macinko

**Affiliations:** 1 Fundação Oswaldo Cruz, Centro de Pesquisas René Rachou, Av. Augusto de Lima 1715-30190002, Belo Horizonte, MG Brazil; 2 Universidade Federal de Minas Gerais, Av. Alfredo Balena, 190 – 30130100, Escola de Enfermagem, Belo Horizonte, MG Brazil; 3 Department of Health Policy, Fielding School of Public Health, University of California Los Angeles, Room 31-235B CHS, Los Angeles, CA USA

**Keywords:** Activities of daily living, Instrumental activities of daily living, Informal care, Formal care, National health survey, Functional limitation, Social inequalities, Socioeconomic position

## Abstract

**Background:**

This study assesses the association between socioeconomic factors and living arrangements with activity of daily living limitations (ADL) and the receipt of informal and formal care among non-institutionalized Brazilians aged ≥ 60 years.

**Methods:**

Data come from a nationally representative survey (the Brazilian National Health Survey), conducted in 2013. Outcomes examined include the number of ADL tasks performed with limitations and number of tasks for which the individual received informal care (provided by unpaid relatives or friends), formal care, or no care. Key exposure variables were years of education and household assets.

**Results:**

Functioning limitations were reported by 7,233 (30.1 %) of 23,815 survey participants. Of these, 5,978 reported needing help to perform at least one ADL task. There was a strong inverse gradient between physical functioning and both education and household assets that was independent of confounders. The provision of care showed an opposite trend, with the wealthiest being more likely to receive help for performing ADL tasks. The receipt of formal care was strongly correlated with highest education (Fully adjusted prevalence ratio [PR] = 1.64; 95 % CI 1.05, 2.58) and with the highest assets level (PR = 2.24; 95 % CI 1.38, 3.64). Living with someone else was associated with provision of care (formal or informal) for those at the lowest and intermediate educational and assets levels, but not for the wealthiest.

**Conclusion:**

Despite worse physical functioning, older Brazilians in worse socioeconomic conditions are much less likely to receive needed help in performing ADL tasks.

## Background

Brazil, the world’s fifth most populous nation, has experienced one of the world’s most rapid rates of demographic aging, a trend that will accelerate during the twenty-first century [1]. Globally, the increasing number of older persons has generated concern among policy makers in part because of the related increase in demand for and cost of long-term care [2–4]. The extent of an individual’s disability is a major determinant of whether or not they require long-term care.

Disability can be defined in several ways [5]. A person’s ability to perform basic activities of daily living (ADL) and/or instrumental ADLs is largely used to assess physical functioning in epidemiological and clinical studies. The first scale includes the most basic activities involved in everyday independent functioning (e.g., bathing, dressing, feeding, etc.). The latter describes activities necessary for adaptation to the environment with emphasis on social activities (e.g., shopping, managing money, etc.). Generally, these measures range from “any difficulty” to perform one of more activities to complete inability to perform them (or comparable gradients). Recent cross-national comparisons have used “any difficulty” as the cutpoint to define a physical functioning limitation [3, 6].

Informal care (that provided by non-paid relatives or friends) is the predominant source of long-term care in many countries such as the United States, West Europe and South Korea [3, 6]. The source of long-term care (that is, whether it is provided informally or through formal (paid) means) is strongly correlated with the availability of family members [3] and on the type of policies and programs offered for supporting the older individuals with ADL limitations [3, 7]. Although there have been a few cross-national comparisons, there is evidence that the source of long-term care is a product of both socioeconomic position and social policies and may vary among countries [7, 8].

Brazil has a national health system (the Sistema Único de Saúde, SUS) designed to provide comprehensive and universal care through decentralized management and provision of health services that are free of charge at the point of delivery [9]. As part of the SUS, Brazil has a national health policy for older adults, which considers people’s functioning [10]. However, this policy does not provide home-based long-term care for older persons. In addition, 26 % of Brazilian citizens have private health plans that allow them to access the private health sector [9], although these plans vary considerably and it is not known how many include long-term care provisions.

There is considerable evidence that many of the social determinants of health, be they income, education, or living conditions, are highly inequitably distributed within Brazil. For example, despite absolute reductions in inequalities in recent decades, the Gini index still remains one of the most unequal in the world (0.53 in 2013) [11]. Socioeconomic disparities in older ages are observed across a range of health conditions, as well as in access to and use of healthcare [12, 13]. There is also a well-documented socioeconomic gradient in older Brazilians’ ability to perform basic ADLs, with wealthier persons experiencing better physical functioning than those in lower socioeconomic groups [12, 13]. However, previous reports were based on older nationally representative surveys (conducted from 1998 and 2008), which contained limited information on physical functioning. The Brazil’s most recent (2013) national health survey is more comprehensive. Results from this survey showed that about 30 % of older Brazilians had “any difficulty” carrying basic and/or instrumental ADLs and that, for those with physical limitations, informal (non paid) care largely predominates (≅80 %), with a smaller proportion receiving formal care (≅ 6 %), about 7 % receiving a combination of both informal and formal care, and approximately 6 % reporting they received no help at all [14].

We used data from the above-mentioned 2013 National Health Survey to examine socioeconomic inequalities associated with ADL limitations and the receipt of home-based long-term care among older Brazilians with functional limitations.

## Methods

### Data source

Data come from the National Health Survey (in Portuguese, *Pesquisa Nacional de Saúde - PNS*), carried out in 2013 by the Brazilian Institute for Geography and Statistics in collaboration with the Ministry of Health. The survey used a complex probabilistic sample, whose methodology is described elsewhere [15]. The survey was representative of the Brazilian noninstitutionalized adult population (≥18 years). Interviews were conducted in 64,348 households (response rate = 94 %) [16]. A random sample of those aged 18–59 years and all persons aged 60 years in the sampled households were eligible for the survey [15]. We analyzed data from all participants aged ≥ 60 years (*n* = 23,815) and, for specific analyses, data from those participants with physical limitations and who reported needing help to perform ADL tasks (*n* = 5.978) (see below).

### Measures and methods

An ADL limitation was defined as any difficulty (some, much or unable) to perform at least one out of six basic (eating, bathing, toileting, dressing, walking across a room, getting in/out of bed) and/or instrumental ADLs (shopping, managing money, taking medications, using transport). For those who reported any difficulty, the survey questionnaire asked, separately for each task, if the respondent had any help to perform the activity, with answers categorized as (1) yes; (2) no, although they actually needed help to perform the task; and (3) no, because they did not need help. For those who answered “yes”, the questionnaire then asked who provided help for each task. Thus, the survey questionnaire assumes that those with no difficulty do not need help for the corresponding ADL task. In the current analysis, the need for help was attributed to those who reported needing any help to perform one or more basic and/or instrumental ADL, regardless of whether or not they actually received such help (corresponding to answers (1) and (2) in the above mentioned questions on receipt of help). Given that the respondent may have received help from more than one person, we considered separately the number of activities for which they received help from non-paid persons (informal care) and for paid persons (formal care).

Our key exposure variables were two important measures of socioeconomic conditions: years of education and household assets. In Brazil, formal education is organized into primary school (1–8 years of school), high school (9–11 years), and higher (i.e. college). For our analysis, and given the distribution among Brazilian older adults, we categorized education into three groups: illiterate, 1–8 years and ≥ 9 years. Household assets were defined by a score (see below) based on the household’s number of color TVs, refrigerators, DVD players, washing machines, landline and cell phones, computers, microwaves, personal vehicles, and the number of bathrooms in the house.

Potential confounding variables in our analysis included age (as a continuous measure) and living arrangements (categorized into lives alone, lives with one person, and lives with two or more persons). These variables are associated with physical functioning and/or caregiving under different contexts [3, 6, 14]. Other potential confounders were gender and whether a proxy responded to the interview.

### Statistical analysis

Principal component analysis [17] was used to create a household assets score based on the items previously described. As the score may range from - ∝ to + ∝, we then divided it into three equal groups where higher scores indicated greater household assets or wealth. Our outcome variables were: number of limited ADL tasks; number of tasks for which the respondent reported needing for help to perform; and the number of tasks for which the respondent received informal care, formal care, or a combination of these. Analyses of the provision of long-term care were restricted to older adults with any ADL limitation who reported needing help to perform one or more ADL tasks.

In bivariate analyses, Pearson’s chi square test (for frequencies) and linear regression (for means) were used to assess the statistical significance of differences across years of schooling and household asset tertiles. Given that our count outcome variables were over-dispersed, we employed negative binomial regression models [18] to examine their multivariable association with education and household assets. All regression models included age (continuous), sex, number of persons living in the household (three categories), whether a proxy responded the interview (yes, no), years of schooling (3 categories) and household assets (3 categories). We mutually adjusted schooling and household assets because they showed only moderate collinearity (Variance Inflation Factor = 1.29). We implemented further stratified analysis by schooling and household assets levels to examine separately the association between living arrangements and receipt of formal and informal care.

To visualize how the relationship between ADL limitations and the lack of help to perform ADL tasks changed according to age and household assets, we fitted separate negative binomial regressions of the number of corresponding tasks to estimate predicted probabilities for each outcome, and then plotted the results.

Because our conclusions did not change when we stratified models by sex, we pooled results and included sex in all multivariate models as a potential confounding factor. All analyses used Stata version 13 (StataCorp LLP, College Station, TX). All estimates incorporated the effect of the sample design and individual probability weights.

## Results

Table 1 presents characteristics of the study sample. From a total of 23,815 participants, 31.8 % were illiterate, 46.5 % had primary (1–8 years) and 21.7 % had higher formal education. The mean age of study participants was 69.8 years (SD = 9.3), 56.4 were women, 14.9 % lived alone, 35.6 % lived with one person and 42.3 % lived with two or more persons. The prevalence of ADL limitations was 30.1 %, ranging from 43.0 % among the illiterate to 29.0 % among those at intermediate education and 13.8 % among those with higher formal education (*p* < 0.001). Other characteristics of study participants by schooling level can be seen in Table 1.

**Table 1:** Socio-demographic characteristics of the study sample, by years of education (National Health Survey, 2013)

		Illiterate	1–8 years	≥9 years	*P* value
Unweighted sample size	23,815	8,245	9,985	5,585	
Age, mean (SE)	69.8 (9.3)	72.0 (16.7)	69.6 (14.2)	67.3 (15.5)	<0.001
Female gender	56.4	57.8	56.1	55.0	0.045
Living arrangements					
Live alone	14.9	14,7	14,7	15.2	<0.001
Live with one person	35.6	33.0	35.8	38.8	
Live with two or more persons	42.3	49.4	45.7	49.5	
Household assets in tertiles					
Lowest	33.1	54.9	28.8	7.5	<0.001
Middle	32.2	30.2	27.4	24.2	
Highest	34.6	12.9	33.8	18.2	
Interview responded by a proxy	32.3	33.6	32.0	30.8	0.075
Any activity of daily living (ADL) limitation^a^	30.1	43.0	29.0	13.8	<0.001
Report of needing help for one or more ADL tasks among those with any ADL limitation	24.5	37.0	22.7	9.9	<0.001

All results are percentages, except where specified. ^a^Any difficulty to carry out one or more tasks. *P* value for differences across educational groups (Pearson’s chi square test and linear regression for differences across frequencies and means, respectively). All estimates take into account the complex sample design and survey weighs

Table 2 shows results of the multivariable analysis of the association between schooling and household assets with ADL limitations, as well as need for and receipt of help to perform ADL tasks. The number of ADL limitations was inversely and independently associated with schooling level (PR = 0.79; 95 % CI 0.71, 0.88 for intermediate and PR = 0.48; 95 % CI 0.39, 0.57 for highest level, relative to those who were illiterate). A similar graded association was found for household asset levels (PR = 0.83; 95 % CI 0.74, 0.93 and PR = 0.62; 95 % CI 0.53, 0.73, respectively). Consistently, the number ADL tasks for which the respondent needed help decreased with both increasing education and household assets. The number of ADL tasks for which the respondent did not receive any help (even though they reported needing it) was strongly and negatively correlated with household assets (PR = 0.59; 95 % CI 0.43, 0.80 for the intermediate and PR = 0.44; 95 % CI 0.29, 0.68 for the highest tertile, respectively), but not with educational attainment. With regards to informal help, those with the highest household assets were less likely to receive this type of care (0.84; 95 % CI 0.77, 0.93). With regards to formal help, strong positive associations were found for both highest educational (PR = 1.64; 95 % CI 1.05, 2.58) and household assets levels (PR = 2.24; 95 % CI 1.38, 3.64).

**Table 2:** Results of multivariable analysis of the association between educational level and household assets with activity of daily living (ADL) limitations, need for and receipt of help to perform ADLs among older Brazilians (National Health Survey, 2013)

	No. of ADL tasks with any limitation^a^	No. of ADL tasks for which help is needed^b^	No. of ADL tasks for which help is needed but not received^c^	No. of ADL tasks for which informal (unpaid) care was received^c^	No. of ADL tasks for which formal (paid) care was received^c^
	PR (95 % CI)	PR (95 % CI)	PR (95 % CI)	PR (95 % CI)	PR (95 % CI)
Unweighted sample size	23,815		5,978		
Years of schooling (vs. illiterate)					
1–8	0.79 (0.71, 0.88)*	0.71 (0.62, 0.81)*	0.92 (0.70, 1.20)	0.93 (0.86, 1.01)	0.87 (0.62, 1.21)
≥9	0.48 (0.39, 0.57)*	0.43 (0.35, 053)*	0.95 (0.68, 1.47)	0.88 (0.76, 1.01)	1.64 (1.05, 2.58)*
Wealth tertiles (vs. lowest)					
Intermediate	0.83 (0.74, 0.93)*	0.80 (0.70, 0.90)*	0.59 (0.43, 0.80)*	0.97 (0.90, 1.05)	1.37 (0.94, 2.00)
Highest	0.62 (0.53, 0.73)*	0.58 (0.48, 0.70)*	0.44 (0.29, 0.68)*	0.84 (0.77, 0.93)*	2.24 (1.38, 3.64)*

^a^Any difficulty to carry out the task. ^b^Letter of tasks for which help was needed to perform. ^c^Letter of tasks for which the respondent needed but did not receive any help, or number of tasks for which help was received by paid/non paid persons (among those with ADL limitation and who needed help to perform one or more tasks.) PR (95 % CI): Prevalence ratios and 95 % confidence intervals estimated by negative binomial regression and adjusted for age (continuous), gender, living arrangements (3 categories), proxy respondent for the interview (yes, no), and mutually adjusted for schooling and household assets. *: *p* <0.05 (log likelihood test) All estimates took into account the complex sample design and survey weights

**Fig. 1:**
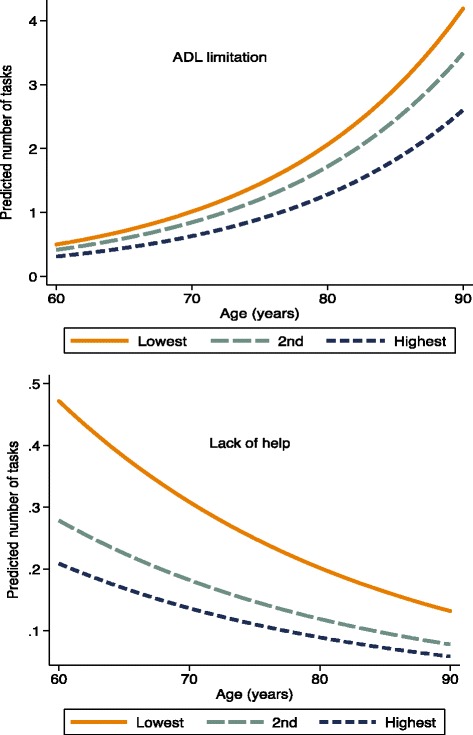
Predicted number^1^ of activities of daily living (ADL) tasks with limitations (top panel) and number of such tasks for which older Brazilians needed help, but did not receive it (bottom panel), by household asset tertile

Figure 1 shows the predicted number of ADL tasks with limitations and the predicted number of tasks for which the respondents did not receive any help by age and household assets in tertiles. The top panel shows a clear stratification that increases with age, with better functioning among the wealthiest. The bottom panel shows an inverse association between receipt of help for ADL tasks and household assets, with the wealthier receiving more care; the gradient of provision of care decreased slightly with age, but still remained largely among the oldest.

Table 3 shows the results of multivariable analyses of the association between living arrangements with the receipt of care, stratified by schooling and household assets levels. Living arrangements were associated with receipt of care among illiterate older adults (PR = 1.27; 95 % CI 1.08, 1.50 for those who live with two or more persons) and among those with intermediate educational level (PR = 1.33; 95 % CI 1.12, 1.58 and PR = 1.47; 95 % CI 1.25, 1.74 for those who live with one and two or more persons, respectively). With regards to household assets, living arrangements were associated with receipt of care among those at the lowest (PR = 1.22; 95 % CI 1.07, 1.39 and PR = 1.34; 95 % CI 1.17, 1.53, for those who live with one and two or more persons, respectively respectively) and among those at the intermediate tertile (PR = 1.25; 95 % CI 1.01, 1.55 and PR = 1.44; 95 % CI 1.18, 1.77, respectively). In contrast, no statistically significant associations were found for those at the highest education or household assets levels.

**Table 3:** Multivariable association between living arrangements and receipt of care^a^ among older Brazilians with activity of daily living (ADL) limitations and who reported needing care for one or more tasks, stratified by educational and household asset levels (National Health Survey, 2013)

	Years of schooling		
Living arrangements	Illiterate PR (95 % CI)	1–9 PR (95 % CI)	≥9 PR (95 % CI)
Live alone	1.0	1.0	1.0
Live with one person	1.14 (0.96, 1.36)	1.33 (1.12, 1.58)*	0.92 (0.67, 1.25)
Live with two or more persons	1.27 (1.08, 1.50)*	1.47 (1.25, 1.74)*	0.97 (0.70, 1.34)
	Household assets tertiles		
	Lowest	Intermediate	Highest
Live alone	1.0	1.0	1.0
Live with one person	1.22 (1.07, 1.39)*	1.25 (1.01, 1.55)*	0.71 (0.46, 1.09)
Live with two or more persons	1.34 (1.17, 1.53)*	1.44 (1.18, 1.77)*	0.76 (0.49, 1.16)

^a^No. of ADL tasks for which the individual received help from unpaid or paid persons (informal or formal care) (unweighted sample size = 5,978). PR (95 % CI): Prevalence ratios and 95 % Confidence Intervals estimated by negative binomial regression and adjusted for age (continuous), gender, and proxy respondent for the interview (yes, no) and mutually adjusted for schooling and household assets. *: *p* <0.05 (log likelihood test). All estimates took into account the complex sample design and survey weights

## Discussion

We examined socioeconomic inequalities in physical functioning and provision of care for older Brazilians in a nationally representative sample. A major finding is that there was a strong inverse gradient between physical functioning with both education and household assets that is independent of important covariates. In contrast, the provision of home-based long-term care showed an opposite trend, with the wealthiest being more likely to receive help for performing ADL tasks. Additionally, the receipt of formal care was strongly correlated with socioeconomic conditions, while socioeconomic stratification was less evident for informal care.

Our findings of a strong gradient across education and household assets on physical functioning is not surprising, given that a number of studies have documented social inequalities on the ability of Brazilian older adults to perform basic ADL tasks [12, 13]. Social disparities in physical functioning in old age have also been documented in high-income countries, with England as an emblematic example [13]. Therefore, the novel finding from our analysis is the strong inverse socioeconomic gradient in the provision of home-based long-term care for older Brazilians.

The receipt of long-term care is influenced by several factors, such as the availability of relatives or friends to provide informal care, cultural norms, and policies to support long term care at home [3, 7]. Previous cross-national studies have showed that the association between socioeconomic indicators and informal and formal care varies across countries [7, 8]. For example, inequalities (defined by material well-being) in the receipt of informal help were found to be greatest in the Netherlands, followed by Great Britain and Italy, and lowest in Belgium. Socioeconomic inequalities in the receipt of formal care are relatively small in these countries with the smallest inequalities in Great Britain and Belgium [7]. Our analyses showed positive relationships between both schooling and household assets with the receipt of formal care, but the association was stronger for the latter factor. Indeed, older adults at the highest tertile of household assets were twice as likely to receive paid help than their counterparts at the lowest wealth tertile. Brazil currently has no national or regional public policies or programs to support ‘in home’ long term care for the elderly [10], as previously mentioned. Thus, paying for long-term care is likely to fall entirely on the individual and his/her family, which explains the strong association between household assets and receipt of formal care.

With regards to informal care, socioeconomic stratification was less evident (with a negative association with highest schooling level). The likelihood of informal care is linked to the availability of relatives or friends to provide such care. In most societies, older persons who live with a relative (child or spouse) are more likely to receive informal care [3, 6, 8]. Our results showed that living arrangements (that is, living with one or two persons and more) was associated with provision of informal care among those in worse socioeconomic conditions. In contrast, living arrangements was not significantly associated with the provision of informal care for the wealthier (which is probably explained by their ability to afford formal care, as previously discussed). This heterogeneity has implications for social policy, as follows. In Brazil, as in other countries, the availability of informal care is a concern because this type of care will likely decrease in the near future as a result of reducing the size of families, increasing numbers of couples without children, and the increased participation of women in the labor market [2, 19, 20]. Our findings of heterogeneity by socioeconomic conditions on the association between living arrangements and the provision of informal care strongly suggest that the impact of the above mentioned demographic changes will be particularly dramatic for those at the intermediate and lowest socioeconomic strata.

This study has strengths and limitations. The main advantage is the large nationally representative population-based sample. This allowed for the first time to quantify the magnitude and the association between socioeconomic conditions and the receipt of informal and formal care among older Brazilians. Another advantage of the study is its internal validity, given that the PNS produced high quality data, with careful preparation of instruments and quality control of data collection and processing [15]. On the other hand, the study has limitations inherent to its cross-sectional nature, and we are not able to make any inference about temporal relationships between source of care and socioeconomic indicators or living arrangements. Further, our analysis did not include an important indicator, income, given that this information was not available when our analysis was implemented. However, income may be less important to those who have retired than household assets. One’s spouse and children are important sources of informal care for older adults [3, 6, 21, 22]. But it was not possible to establish the specific relationship of informal caregivers to the older adult requiring care, which is another limitation of this analysis.

## Conclusion

The results of the current analysis reveal important social disparities in physical functioning of older Brazilians, with worse performance among those at the lower educational and household assets levels. Importantly, despite worse physical functioning, older people with worse socioeconomic conditions were much less likely to receive help to perform ADL tasks. Given recent demographic changes, Brazil is likely to experience an even greater number of aged persons with physical limitations alongside a decrease in the availability of informal caregivers. If these trends continue, social disparities in the provision of care for older Brazilians are likely to continue to widen.

## Abbreviations

ADL: Basic activities of daily living
IADL: Instrumental activities of daily living
OR: Odds ratio
PNS: National Health Survey (in Portuguese, *Pesquisa Nacional de Saúde*)
SEP: socioeconomic position
SUS: National Health System (in Portuguese, *Sistema Único de Saúde*)
